# American Academy of Dental Science

**Published:** 1894-05

**Authors:** 

**Affiliations:** American Academy of Dental Science


### AMERICAN ACADEMY OF DENTAL SCIENCE.

THE regular monthly meeting of the American Academy of Dental Science was held at Young's Hotel, Boston, on Wednesday evening, January 3, 1894, at six o'clock, President Smith in the chair.

*President Smith.*—Gentlemen, we come now to the subject of the evening, "Crown- and Bridge-Work." The subject will be opened by Dr. Cecil P. Wilson.

(For Dr. Wilson's paper, see page 303.)

### DISCUSSION.

*Dr. Wilson.*—Dr. Moffatt, the other day, as a matter of experiment, coated several teeth with enamel, put them in a Parker gas furnace, and baked them eight minutes, subjecting all to exactly the same conditions. The kinds of teeth experimented on were as follows:

(1) Howard & Tucker. The body of this tooth is composed of eight pennyweights of silex, twelve pennyweights of spar, and twenty grains of clay.

(2) S. S. White Dental Manufacturing Company.

(3) Johnston & Lund.

(4) C. Ash & Son.

(5) The old Stockton tooth.

The tooth from C. Ash & Son apparently made the poorest showing. It was all puffed up, and had entirely lost its original shape and color. It looked like a lady's button more than anything else. The teeth made by the S. S. White Dental Manufacturing Company and Johnston & Lund came out very well. The old Stockton tooth, so much used in years gone by, did fairly well, but the gum enamel was faded and looked very glossy. The old Howard & Tucker tooth stood the test best of all. I have no right to say that the Ash tooth is any less durable in the mouth than the other teeth.

*Dr. Williams.*—There are two points on which I think something might be said. I was glad to hear Dr. Wilson say that too soft gold must not be used where there is any strain. I found some time since that the finer gold is too soft. I tried those bands that are graded in sizes and sold by the S. S. White Dental Manufacturing Company, but, after getting them fitted, I found in two or three cases that they stretched, and so gave them up and have since made my own bands of stiffer gold.

I have not heard mentioned in reference to bridges one cause of irritation of the roots, which seems to me a very important one. This irritation is generally supposed to result from the bands or crowns being too far up. There is another cause, and it was this which led us, in 1844, to discourage extensive bridge-work permanently fastened to several roots. If you take an impression of the strongest set of teeth in the mouth of a man in good health and medium age, and make an accurately-fitting plate, and let the patient wear it a day or two and then take it off and leave it off for a day, when he comes to put it on again he finds it goes on a little hard at some particular point. If the plate is left off two or three days it does not fit as it should. What is the reason? There is in the average set of teeth a little swinging, swaying movement, or life, as it were. Each tooth is changing its position with, however, only a very slight movement, and has a different relation to the other teeth from day to day, and perhaps during the same day. That natural movement—you might say elasticity—of the row of teeth, if rigidly held in check by a metal plate fastened to the teeth, produces a strain which is often the cause of premature loosening of the roots. The teeth do not have freedom of exercise, so to speak, when held firmly by a rigid fastening. If that fact were taken into account I think there would be a little less use made of extensive pieces of bridge-work permanently fastened in the mouth. The first piece of work I had given me to do in order to get my hands trained, when I was studying with Dr. Keep, was to carve out of sea-horse material, such as had been used for artificial teeth in the early stage of the art, six front teeth which it was intended to pivot to several roots. This pivoting was, of course, open to the objection of being firmly held, and that was a part of the experience which led me to see the discomfort of extensive permanent cases of bridge-work. The principal is all right if you apply it judiciously, but I prefer, if possible, to have such appliances removable.

*Dr. Brackett.*—It has been said that one of the most delicate and graceful compliments that an author can pay a friend is to give him a copy of his book containing a specimen of his writing, and that in the same way it is evidence of high esteem for a painter or an artist of any kind to present something that he has himself done in the line of his work. And so also social economists and students of moral philosophy say there is no high and true charity that does not involve the giving of something of one's own self; that the mere bestowal of money or something of that sort that may be done carelessly, without taking an active interest, is not true charity. It seems to me that the essayist to-night has in the highest and best sense put before us that which is most complimentary and helpful to us and most honorable to him,—that he has been rarely good to us, and of that in itself I wish to express my high appreciation, and I am sure that every one in the room has the same feeling.

Further than that, I wish to express my admiration of the general good sense and moderation of the paper. The purport of it is that bridge-work in itself has great merit when judiciously used, skilfully constructed, and properly applied. And this is decidedly my own feeling out of some experience not large, but very gratifying. I think we find generally that it is along these lines that the value of all meritorious things lies. Whether we contemplate some particular kind of filling-material or some ingenious contrivance, a very great deal of the success lies in the good judgment and wise discrimination of cases in which it is used, and the skilful adaptation of means to ends. I am especially gratified to have been present this evening, and to have heard a paper which seems to have put before us the man himself in the most generous, kindly, and brotherly way.

*President Smith.*—I want to ask Dr. Wilson what was his method of fitting clasps to the supporting teeth?

*Dr. Wilson.*—I first took an impression of the gold crown in plaster, into which I poured fusible metal. To this die I fitted accurately a band of twenty-carat gold, No. 30 gauge. I then made another band of clasp metal and soldered the two together. This made a perfectly-fitting clasp. I do not think, however, I should recommend this sort of clasp for all kinds of cases. In this case of mine the clasps slip into their places very easily. In cases, however, where the teeth are irregular in shape, and the clasps must be sprung into place, the ordinary clasp metal alone must be used.

*President Smith.*—Do you solder all the teeth and the clasps at one bedding, or in parts, and then unite the parts?

*Dr. Wilson.*—I think the proper way to construct a piece of this kind is to make it in sections. The first section included the four incisors, then the other two sections were made, an articulation taken, and the teeth soldered together. The case was made in three sections, just as though it was three different pieces of work; each piece was soldered separately and they were placed in the mouth, an impression taken of the whole, and all joined together.

In making the case that I have in my mouth I made one complete plate, as I thought it would save a little time. The result is, I can feel that the fit is not so accurate as it would have been if I had made it in sections. I venture to make the assertion that it is absolutely impossible for any man, no matter how skilful he may be, to so heat up a plate that it will fit just as it did before the heating took place. There must be a little warping, and more or less expansion and contraction during the heating-up process. These molar blocks were backed up and soldered separately. The backings were well covered with solder, so that when they were finally placed in the investment it required but a little solder to join the pieces together.

*Dr. Williams.*—I would like to ask Dr. Wilson if there would be any objection if, in striking up this plate, he strikes it up in one piece and then divides it and makes each part separately?

*Dr. Wilson.*—There is not the slightest objection to striking up the whole thing at once and then dividing it, but I happened to strike it up separately.

*Dr. Williams.*—Of course, if they were to be struck up in one piece, they would require another fitting after the dividing.

*Dr. Wilson.*—Certainly. I might say still further that this is not Dr. Richmond's method of doing this kind of work. He uses pure gold and burnishes it down either on a metal or a plaster cast, and in that way avoids the necessity of striking up a plate. He is very skilful and does his work very nicely.

*Dr. Moffatt.*—I would suggest that Dr. Wilson explain the method of taking the last impression of the different pieces which are to be joined together in the mouth with perforated tin.

*Dr. Wilson.*—If I wanted to join two pieces, I should wax them together on the model and place them in the mouth. In order to get an accurate impression of these teeth, I use a piece of perforated tin such as is used in the making of kerosene lamps or coffee-strainers. This idea I got from Dr. Richmond, of New York, though I do not do it exactly as he does. He uses thin brass, the holes in which are quite large. You can bend the tin in any shape, and the holes allow the surplus plaster to go through, and yet the tin holds the plaster in place.

*President Smith.*—The perforated tin serves as a cup, and you bend it into different forms.

*Dr. Moffatt.*—You can get the perforated tin from the Dover Stamping Company, on North Street, Boston. There are various thicknesses, and it takes but a minute to bend it into any desired shape, and fasten any cuts or corners with a little soft solder.

*Dr. Baker.*—I would like to ask Dr. Wilson how he grinds the tooth, or shapes the root, to make the cap go over it. What method or what instruments does he use?

*Dr. Wilson.*—I use a corundum wheel.

*Dr. Baker.*—But in cutting under the gum,—in making the parallel surfaces on the molar and bicuspid in the case you exhibited,—do you use root-trimmers such as are sometimes used for putting on a Richmond crown?

*Dr. Wilson.*—The conditions are favorable in the case presented for a perfectly-fitting crown. You will remember I intimated that when I said it was possible to make a perfect impression of these teeth. If they were bell-shaped, a perfect impression would have been impossible.

*Dr. Potter.*—What instrument do you use to trim the edges off above the gum, so that the cap will fit all around? The collar is supposed to go up under the gum.

*Dr. Wilson.*—I use a scaler very much like a scythe. You can get down under the gum with that and cut off the edges. And then I use a fissure bur a good deal.

*Dr. Ainsworth.*—This is a very interesting subject, and opens a wide field for discussion. I wish, in the first place, to thank Dr. Wilson for the practical illustration of it. His testimony must be of value. As to the preparation of the root for the band, I am not aware that I have any different methods from those in use by others. In cases where the root is badly decayed or unfortunately broken, it becomes a difficult and many times a trying ordeal for the patient and operator.

In typical cases, however, of the upper bicuspids, where the crown is cut off and everything favorable, I use a stiff shank hoe-scaler for the larger part of the work. These instruments, as they usually come to us, are susceptible of much improvement by a little careful grinding on a small corundum wheel. The S. S. White Dental Manufacturing Company has recently brought out an obtuse-angle scaler which seems much nearer the acme of perfection than anything heretofore at hand. The operation, if painful, is made much more bearable by frequently dipping the point of the scaler in strong carbolic acid, aiming to interfere as little as possible with the peridental membrane both in the preparation of the root and the adjustment of the band. I usually make the band from a piece of plate while the patient is in the chair, bevelling the edges as they are brought together and soldering. The size is easily determined by tying a ligature around the corresponding tooth. For instance, supposing the tooth to be crowned is a first bicuspid on the right side. If the first bicuspid on the left is in good condition, I would pass a ligature around that, cut, and remove, and thus have a perfect measurement for the required band. A tapering mandrel is the next important auxiliary. I have a very convenient one in a small steel rod that belongs to my great-grandmother's spinning-wheel; it is, perhaps, eighteen inches long and of a convenient diameter. If the band is a trifle small, it is easily enlarged by placing on the mandrel and tapping it gently with a small hammer. If too large, the edge under the gum-line is easily made smaller by the use of the crimping-plate designed for that purpose.

Dr. Wilson spoke of the difficulty of fitting a gold crown accurately under the gum. I have found much satisfaction in constructing a molar crown in this way: Prepare the root as best you can for the reception of the band, which should be wide enough to represent the height of the finished crown. Shape and fit the band nicely, crimping the edge which goes under the gum so that it will take a firm hold of the root at that point. Then select a porcelain top, such as are on the market, with two small pins on the under side, and fit that into the band, and grind to a proper occlusion with the opposing tooth. When this has been done place the band firmly on the roots, applying the dam, and set the top in cement, allowing the patient to close the teeth and bring it to place. This gives a crown less guilded in appearance and in every way satisfactory, and there is the advantage of being able to see exactly how a crown band fits under the gum.

*President Smith.*—As Dr. Baker asked the question of Dr. Wilson in regard to trimming roots preparatory to putting on a band, I would like to ask him if he has any particular method for trimming roots?

*Dr. Baker.*—The reason I asked the question was because I always had great difficulty in doing that part of the work, and, seeing these teeth adjusted so nicely, I thought he must have some special way of doing it. I have tried the various instruments for sale at the dental depots and they work fairly well, but I would like something better if it were possible to get it.

*President Smith.*—I would say that Mr. Heald, who manufactures burs in New York, takes your old broken burs and makes a fine-pointed fissure bur having the shape of a fine cone. This is a very nice thing for preparing a root for the reception of a band. I have read somewhere of a mechanism for this purpose which is attached to the dental engine. It consists of a file with an up-and-down motion.

*Dr. Wilson.*—I have no doubt that a great many gentlemen here can cite cases where all-porcelain teeth have been worn, doing service for a number of years. I recall one case of a lady who wore a porcelain tooth for twenty years; it was secured by an old-fashioned wooden peg set in rubber cement. That, of course, is an exceptional case. There is an instance in my own family where an upper left lateral incisor has been worn for seventeen years. It was a plate-tooth, with gold backing and pin, like a Richmond crown with the exception of the band. It has been reset two or three times during that period and has done good service. Because of these two excellent results, however, I do not consider that the all-porcelain tooth is best. Dr. Tucker, Dr. Williams, or Dr. Preston can show you a great many excellent gold fillings that were put in without the rubber dam and all the other accessories of the present day; but it does not follow that that is the best way of placing in a filling. It seems to me that the Richmond method is the best which is known to-day of fitting on a crown. The difficulty of the operation is a disadvantage, and I sincerely wish I could find some other way of crowning teeth that would be less exacting and yet be fully as serviceable.

There is one thing I would like to ask some information about. I have made crowns in the old-fashioned way with gold or platinum pins, and set them in the teeth with gutta-percha. I have wondered a good many times whether the gutta-percha does not swell and split the roots. I cannot say that I ever saw a root with a crown on it that I could absolutely say was split because of the gutta-percha, but I have a case in mind where the root was filled with gutta-percha and where there was no occlusion, and it was split directly open, and I could not attribute it to any other cause than the swelling of the filling.

*Dr. Williams.*—How large a canal was it, and what class of gutta-percha did you use,—the soft or the dense kind?

*Dr. Wilson.*—I don't remember much about it now. It was some years ago, and I simply recall the fact of finding the tooth gaping open. It must have been the soft kind, because we did not have at that time the dense gutta-percha such as is now used. You understand there was no occlusion and the root had no crown at all on it.

*Dr. Williams.*—The softer kind is elastic and will expand, and is especially liable to do so during mastication of food, whereas the denser kind is not so liable to spread. There is danger of capping pulps with this soft kind on account of its yielding tendency. Dr. Wilson spoke of the durability of teeth set in the older style. I remember a case of an elderly lady who had a porcelain tooth which was occupying the place of the upper right canine, the crown of which had been accidentally broken off. The root had been crowned in the old-fashioned way twenty-eight years before with a simple wooden pin,—hickory, I think it was,—but the crown was nicely adapted to the root and there was very little strain against it. Every circumstance was favorable to the tooth, and the lady wanted me to promise her that it would last thirty years. I put in the same tooth with a new pin, and seven years after that it was still good, so that I know she got thirty-five years use of that old-fashioned tooth.

*Dr. Cooke.*—There was one point which Dr. Ainsworth brought out that I did not quite agree with. He spoke of having the band crimped, so that the lowest part of the band would take a firm hold of the root. I tell the students at the dental school that if they wished to fit a ferrule to a fishing-pole, they would want to have that ferrule fit at all points; and so you must have your band come down parallel with the root and get a substantial friction hold.

Dr. Wilson spoke of the disadvantage of using crowns already in the market, and I thoroughly agree with him. I also agree with him in regard to what he said about the necessity of fitting the crown in the mouth instead of to a model. Unless I can have the band tight enough so that the finest Donaldson broach cannot pass between it and the root, I do not think I have got the band to fit as it should. In regard to irritation from a band, there is no reason why we should have it, provided the crowns are fitted with a reasonable degree of accuracy. When I have heard of a case where there was irritation, I have always made up my mind that the trouble lay in the crown not fitting.

There is one point that I have not heard referred to, and that is the building up of the thin edge of a root with amalgam. We sometimes have only a small portion of the root on which we can secure our band, and when a little absorption goes on, the gum recedes and gives an opportunity for the lodgement of food, and then decay begins underneath. In such cases I build up with amalgam and polish the edges, and then carry an all-gold crown over all.

*President Smith.*—I wish to say, in regard to the possibility of gutta-percha splitting the roots, that I am of the same opinion as Dr. Wilson. I have reached my conclusion from observation, and so strong is that conclusion in my mind that I do not fill canals with gutta-percha, but set the pin with some of the cements.

In measuring for a band I use a silk ligature, carrying it around the root once and cutting at the point of intersection. I never cut the band any longer than my measure, and generally a trifle less. I never lap a band. In that way I think I can get a more accurate fit.

*Dr. Clapp.*—I did not think of saying anything to-night, but Dr. Cooke spoke of dressing up with amalgam badly-decayed roots, and that awakened tender memories of my own case. A good dentist of this town has been operating on some bicuspids in my mouth. They were badly broken down and it was necessary to do something to strengthen them before crowns could be put on. I am unfortunate enough to have gums so sensitive that there are not adjectives enough in the English language to describe the condition. The work on these roots left memories that will be lasting. The method which that gentleman used was to fit very thin platinum bands to these roots. The bands extended a little way below the gum and were fastened to the roots with cement. Howe posts were screwed into the roots and also set with cement, and then the bands were filled with amalgam. This operation was done about a year ago, and when the gentleman gets time, and I get time, I hope gold caps can be put on without much further suffering on my part. The bands are to stay there, and the gold caps, when they are made, are to be fitted around these bands; and I trust, when Dr. Stevens puts the gold caps on, he will not have to interfere with the gums as much as he did last time, although he did it with the greatest possible care.

*Dr. Andrews.*—I would like to ask Dr. Stevens what amalgam he used in filling in those bands?

*Dr. Stevens.*—Blackwood's White Alloy. In regard to getting the size of the root with a string, I think that method inaccurate. I would not attempt to get it that way. The method which I always use in getting the size is to carefully trim my root in the way all the rest of you do, and then take a piece of German silver or copper,—anything that is thin,—make a band of that, fit it over the root, leaving it a little large. I carry the band to place, pass a ligature twice around the same, and, holding the two ends firmly in one hand, burnish the band carefully to place. Tie the ligature, remove the band, and cut open where it laps. In that way I get a very exact pattern, both in size and shape, by which to cut my gold, which is made no larger, and perhaps a trifle smaller, than the pattern. I never lap my bands, always butt and solder them. Of course, if the band is a little loose, I cut and solder it again.

Speaking of irritation of the gum, I have seen many cases of crowns put on with gold bands, perhaps twenty-eight gauge, and not bevelled at all, but the full thickness of the band was driven up under the gum, and it is not surprising to me to find such bands causing irritation.

*Dr. Andrews.*—There is one important thing it would be well to speak about, and that is the cutting away of a considerable surface of a live tooth. It is well known that when dentine is reached, the exposed part of the dentine is irritated, and this irritation sooner or later may result in a pathological condition. There is a question whether it would not be best to kill the pulp at once. I have heard recently of the supporting teeth of a bridge loosening and coming out on account of this change. Is it safe to trim away a great deal of a live tooth to put a cap on?

*Dr. Wilson.*—In reply to Dr. Andrews, I would say that the grinding of the teeth has not in my case been followed by any disagreeable consequences. When I take the bridge off and brush the natural teeth with cold water, I notice a little difference in temperature.

WILLIAM H. POTTER, D.M.D.,
*Editor American Academy of Dental Science.*

